# Proteomic Profiles of Seminal Plasma in Individuals with Secondary Infertility: Insights into the Involvement of Oxidative Stress

**DOI:** 10.3390/jcm15031173

**Published:** 2026-02-02

**Authors:** Raneen Sawaid Kaiyal, Sromona D. Mukherjee, Manesh Kumar Panner Selvam, Aaron W. Miller, Sarah C. Vij, Scott D. Lundy

**Affiliations:** 1 Glickman Urological and Kidney Institute, Cleveland Clinic Foundation, Cleveland, OH 44106, USA; vijs@ccf.org (S.C.V.); lundys@ccf.org (S.D.L.); 2 Department of Cardiovascular and Metabolic Sciences, Cleveland Clinic Foundation, Cleveland, OH 44195, USA; mukhers@ccf.org (S.D.M.); millera25@ccf.org (A.W.M.); 3 Department of Urology, Tulane University School of Medicine, New Orleans, LA 70112, USA; mpannerselvam@tulane.edu

**Keywords:** seminal plasma, proteome, oxidative stress, secondary male infertility, bioinformatics

## Abstract

**Background/Objectives**: Male infertility, including primary and secondary infertility, is significantly influenced by oxidative stress, which disrupts sperm function and fertility. Seminal plasma, a protein-rich fluid essential for sperm protection and function, represents a valuable source for identifying biomarkers through proteomic analysis. While previous studies have explored seminal plasma proteins in fertility, the specific proteomic changes associated with oxidative stress in secondary infertility remain unclear. This study aimed to characterize these alterations by analyzing seminal plasma from three groups: men with secondary infertility, fertile donors with high oxidative stress, and fertile donors without oxidative stress. **Methods**: Pooled semen samples from each group underwent quantitative proteomics analysis using advanced mass spectrometry, with subsequent bioinformatic analysis using tools like DAVID, STRING, and IPA for identifying differentially expressed proteins (DEPs). **Results**: Quantitative proteomic analysis identified 377 DEPs in secondary infertility and 523 DEPs in fertile donors with high oxidative stress compared to controls. Bioinformatic analysis revealed seven shared pathways, including acute-phase response signaling, organismal injury, cellular movement, cell-to-cell signaling, free radical scavenging, immune cell trafficking, and Hematological system development. Notably, C3 and SERPINA3 exhibited significant alterations, along with proteins involved in sperm motility, capacitation, and fertilization, suggesting their potential roles in impaired fertility. **Conclusions**: These findings underscore the link between oxidative stress and secondary infertility and highlight specific seminal plasma proteins as potential biomarkers and therapeutic targets for diagnosing and treating male infertility.

## 1. Introduction

Male infertility constitutes a significant cause of couple infertility, with an estimated 50–60% of cases being attributed to a male factor either as the primary or contributing cause [1], defined as primary or secondary infertility, respectively. Male primary infertility refers to patients that have never fathered a child, whereas secondary infertility refers to patients that achieved pregnancy in the past but are currently unable to achieve one [2]. Secondary infertility is believed to constitute a variable percentage of male infertility, causing a large degree of disability and stress in couples [3]. Multiple factors, including varicocele, infection, smoking, obesity, and exposure to high temperature and hazardous materials, are associated with secondary male infertility, and oxidative stress is proposed as the underlying mechanism [4].

Oxidative stress occurs when the production of reactive oxygen species (ROS) surpasses the antioxidant capacity within cells, adversely affecting sperm characteristics and leading to DNA damage, mitochondrial dysfunction, and apoptotic-like changes, all of which contribute to male infertility [5]. Sperm cells are particularly susceptible to ROS owing to high levels of polyunsaturated fatty acids in both the membrane and cytoplasm and their limited ability to counteract oxidative stress; consequently, they rely on seminal plasma for anti-oxidative protection [6]. Seminal plasma, rich in proteins and other molecules such as sugars and lipids, is crucial for maintaining sperm function and its fertility potential [7]. This protein-rich composition also makes seminal plasma a valuable biological fluid for biomarker discovery and a target for proteomics and bioinformatics analyses [8].

Previous research has provided important insights into male infertility through proteomic analyses of seminal plasma, but it has not thoroughly examined oxidative stress as a mechanistic factor in secondary infertility [9]. In contrast, our study specifically focuses on the proteomic changes in seminal plasma associated with oxidative stress in secondary infertility. By examining how oxidative stress impacts protein composition in cases of secondary infertility, we aim to identify specific biomarkers and therapeutic targets that could provide preventative strategies for men at risk of oxidative damage. This focused investigation of oxidative pathways represents a novel approach, offering direct insights into a crucial factor in secondary infertility and distinguishing our research from previous proteomic studies that did not comprehensively address this mechanism.

To date, limited research has focused on these protein alterations, leaving a critical need for detailed insights that can improve understanding and management of secondary infertility in affected men. We hypothesized that oxidative stress is associated with secondary infertility; therefore, we aimed to elucidate the proteomic modifications in the seminal plasma of individuals with concomitant oxidative stress and secondary infertility. We believe that our findings will aid in advising patients vulnerable to oxidative stress to take appropriate measures to address infertility in the future.

## 2. Materials and Methods

### 2.1. Ethical Statement

For this study, we used proteomic datasets originally procured from prior studies conducted at our Andrology Center, Cleveland Clinic, Cleveland, OH, USA [10,11]. The proteomics data utilized were derived from our proteomic project on male infertility in which we conducted multiple proteomic analyses on infertility patients with various etiologies, such as varicocele, primary infertility, and secondary infertility [10,12].

In this study, we chose to focus on a group of patients with secondary infertility. Our hypothesis evolved from preliminary findings in our broader proteomic analysis. All participants, including patients and donors, signed informed consent in accordance with the Declaration of Helsinki. The samples used for proteomic analysis followed the Minimum Information about a Proteomics Experiment (MIAPE) guidelines of the Human Proteome Organization’s Proteomics Standards Initiative (HUPO-PSI) for reporting proteomics studies [13].

### 2.2. Sample and Data Collection

Semen samples were collected from three defined groups:

#### 2.2.1. Patients with Secondary Male Infertility (*n* = 10)

- Secondary infertility was defined as the inability to conceive a second or subsequent child after one year of unprotected intercourse.
- Exclusion of female factor based on normal gynecologic evaluation, ovulatory function, and absence of known uterine or tubal pathology.
- Inclusion criteria included men with mild (>10 × 10^6^ to ≤15 × 10^6^ sperm/mL) to moderate oligospermia (>5 × 10^6^ to ≤10 × 10^6^ sperm/mL) or asthenozoospermia, teratozoospermia and confirmed oxidative stress levels (ROS > 93 RLU/s).
- Exclusion criteria included chronic diseases, varicocele, azoospermia, or severe oligospermia (≤5 × 10^6^ sperm/mL).

#### 2.2.2. Fertile Donors with High Oxidative Stress (*n* = 10)

- Healthy men who had fathered at least one child within the last two years.
- Displayed elevated seminal oxidative stress levels (ROS > 93 RLU/s).
- Exclusion criteria included, chronic diseases, varicocele, azoospermia, or severe oligozoospermia.

#### 2.2.3. Control Group of Fertile Donors Without Oxidative Stress (*n* = 10)

- Healthy men who had fathered at least one child within the past two years.
- No history of oxidative stress (ROS < 93 RLU/s).
- Exclusion criteria included conditions such as chronic diseases, smoking varicocele, infections, leukocytospermia, hormonal imbalances, BMI > 30 kg/m^2^, azoospermia, or severe oligospermia.

### 2.3. Semen Analysis and Assessment of Oxidative Stress and DNA Fragmentation

Semen samples were collected and analyzed in accordance with the World Health Organization (WHO) guidelines [14]. Participants were instructed to maintain an abstinence period of approximately five days prior to sample collection. All samples were allowed to liquefy at 37 °C and were analyzed within one hour of ejaculation. Semen parameters were assessed using a disposable Leja sperm counting chamber (Spectrum Technologies, Healdsburg, CA, USA).

In addition to standard semen analysis, we evaluated ROS levels and sperm DNA fragmentation (SDF). ROS levels were quantified using a chemiluminescence assay, employing a validated cutoff value of 93 relative light units per second (RLU/s) to distinguish between high and low oxidative stress, as previously described [15,16]. SDF was assessed using the terminal deoxynucleotidyl transferase-mediated fluorescein-dUTP nick end labeling (TUNEL) assay, a well-established method for detecting DNA strand breaks at the single-cell level and providing insight into sperm nuclear integrity [17].

**Figure 1:**
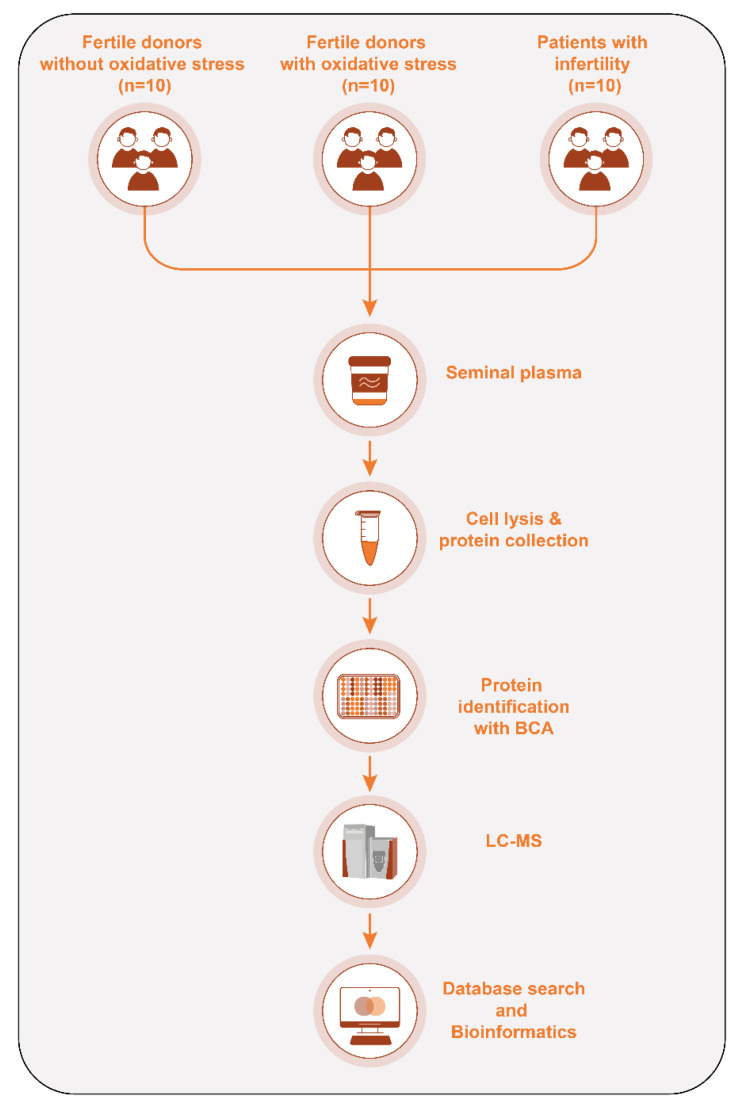
Study flowchart: semen samples were collected from three groups of participants: healthy fertile donors ROS-negative control group (*n* = 10), fertile donors with ROS-positive (*n* = 10) and patients with secondary infertility (*n* = 10). Seminal plasma was separated and subsequently preserved at −80 °C for proteomic evaluation. Pooled samples from the three groups were used for proteomic analysis. The samples were mixed with SDS-PAGE buffer and subjected to 1D-PAGE in triplicate runs to address technical variation. Proteomic profiling of seminal plasma was conducted using a Finnigan LTQ linear ion trap mass spectrometer (LC-MS/MS system). Bioinformatics analysis using multiple software applications.

Following semen analysis, seminal plasma was separated by centrifugation at 13,000× *g* for 20 min at room temperature (24 °C) and stored at −80 °C for subsequent proteomic analysis. The detailed methodology and experimental workflow are illustrated in Figure 1.

### 2.4. Proteomic Sample Preparation and Mass Spectrometry

Seminal plasma samples were thawed and centrifuged at 3000× *g* for 30 min to remove residual cells and debris. Protease inhibitors (Roche, Indianapolis, IN, USA) prepared in PBS were added in a 1:1 ratio to prevent protein degradation. Total protein concentrations were quantified using the BCA assay (Thermo, Rockford, IL, USA). Equal protein concentrations (15 mg/mL) were pooled from each group (*n* = 10 per group) to normalize for inter-sample variability. Seminal plasma proteins were separated using 12% SDS-PAGE gels under denaturing conditions. Gels were stained with Coomassie Brilliant Blue R-250 to visualize protein bands, which were subsequently excised for in-gel trypsin digestion prior to LC-MS/MS analysis. Gel bands were excised, reduced with dithiothreitol, and alkylated with iodoacetamide. Each band was cut into six fragments and digested with trypsin (10 ng/µL) in 50 mM ammonium bicarbonate overnight at 24 °C. Peptides were extracted in 10% acetonitrile with 5% formic acid, evaporated to <10 µL, and reconstituted in 30 µL of 1% acetic acid.

Peptide mixtures (5 µL) were loaded onto a reversed-phase C18 capillary column (Phenomenex, Jupiter, FL, USA) and separated by HPLC at a flow rate of 0.25 µL/min. Eluted peptides were analyzed using a Finnigan LTQ Orbitrap Elite hybrid mass spectrometer system (Thermo Fisher Scientific, Waltham, MA, USA).

### 2.5. Protein Identification and Validation

MS/MS spectra were searched using Proteome Discoverer (v1.4.1.288), incorporating Mascot, Sequest, and X! Tandem (CYCLONE version). Results were validated with Scaffold (v4.0.6.1). Peptides were accepted at ≥95% confidence and proteins at ≥99% confidence with an FDR < 1% and a minimum of two unique peptides. Protein probabilities were calculated using the Protein Prophet algorithm [18,19]. Functional annotation was enriched using GO terms retrieved from NCBI (accessed 21 October 2013).

### 2.6. Protein Quantification and Differential Expression

Protein abundance was estimated using spectral counts (SpCs), normalized by the NSAF method to correct protein length and total spectra per sample [20,21]. DEPs were identified using statistical testing and fold-change thresholds. Proteins were stratified into four abundance categories (very low, low, medium, high), with criteria adjusted per category to reduce errors in quantifying low-abundance proteins. The validity of this approach was confirmed through internal controls.

### 2.7. Bioinformatics and Pathway Analysis

Initial protein annotation was performed using GoTerm Finder (https://go.princeton.edu/cgi-bin/GOTermFinder, accessed on 15 June 2024) and UniProt (https://www.uniprot.org, accessed on 15 June 2024). DAVID (https://david.ncifcrf.gov, accessed on 15 June 2024) was used for functional classification into GO categories, including molecular functions, cellular components, and biological processes. STRING (https://string-db.org, accessed on 15 June 2024) was used to build protein–protein interaction networks and to perform enrichment analysis. Further interpretation of canonical pathways, regulatory networks, and disease associations was conducted using Ingenuity Pathway Analysis, IPA version 2025 (Qiagen, Redwood City, CA, USA).

### 2.8. Statistical Analysis

#### 2.8.1. Overview

All statistical evaluations, including data preprocessing, hypothesis testing, and adjustments for multiple comparisons, were performed using MedCalc (v17.8) and R (v4.1.0).

#### 2.8.2. Sample Size Justification

Sample size was calculated based on previous proteomic research [10], aiming to detect a 1.5-fold difference in protein expression with 80% power and α = 0.05. A sample size of *n* = 10 per group was deemed adequate for both differential expression and enrichment analyses.

#### 2.8.3. Proteomic Data Preprocessing

- Normalization: Protein input was standardized to 15 mg/mL. Spectral data were normalized using NSAF.
- Transformation: Log2 transformation was applied to normalized values to stabilize variance.
- Missing Data: Proteins with >50% missing values were excluded. For others, missing values were imputed using a low-abundance method based on distribution-derived constants.
- Testing: Two-sample *t*-tests were used for group comparisons. FDR was controlled using the Benjamini–Hochberg method.
- Degrees of Freedom: Calculated as (n_1_ + n_2_ − 2) for each comparison.

#### 2.8.4. Assumption Testing

Normality was assessed using Shapiro–Wilk and Kolmogorov–Smirnov tests. When violated, log2 transformation or non-parametric tests (Mann–Whitney U) were used. Levene’s test evaluated variance homogeneity; Welch’s *t*-test was applied when variances were unequal. One-way ANOVA was used where appropriate.

## 3. Results

### 3.1. Comparison of Semen Parameters, ROS Levels, and SDF Among Study Groups

Table 1 summarizes the results of semen analysis, SDF, and intracellular ROS testing across the three groups. The control group of healthy fertile donors with ROS-negative status exhibited normal semen characteristics, and low levels of ROS and SDF. In contrast, both study groups exhibited normal values for sperm concentration and morphology, but significantly decreased motility and elevated ROS and SDF levels.

### 3.2. Proteomic Changes in ROS-Positive Fertile Donors and Secondary Infertility Patients

**Figure 2:**
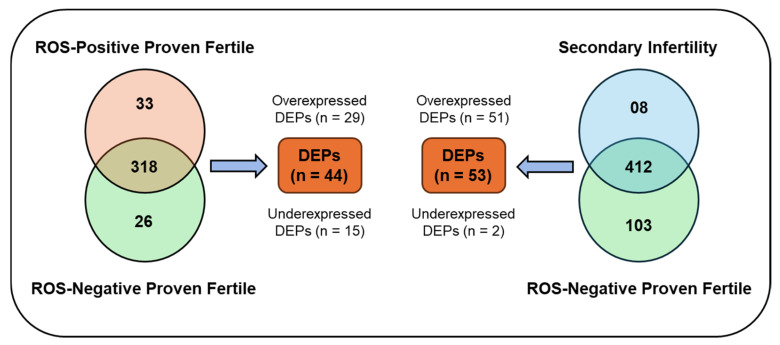
Summary of proteins distribution: number of identified proteins in healthy fertile donors ROS-negative control versus fertile donors. ROS-positive and healthy fertile ROS-negative versus secondary infertile patients.

Distinct proteomic analyses were conducted on the pooled samples from both groups. One comparing a control group of healthy, fertile, ROS-negative donors with fertile, ROS-positive donors, and the other comparing healthy, fertile, ROS-negative donors with patients diagnosed with secondary infertility. Figure 2 summarizes the distribution of proteins. In the comparison between the group of healthy, fertile, ROS-negative donors and the ROS-positive fertile donor cohort, 377 proteins were identified. Of these, 44 proteins exhibited differential expression between the two groups, with 29 being overexpressed and 15 underexpressed (Table S1). Eighteen proteins were classified as highly abundant, 60 as medium abundant, 83 as low in abundance, and 216 as very low in abundance. In contrast, 523 proteins were identified in the group of patients with secondary infertility and high oxidative stress in the semen. Among these, 53 proteins were differentially expressed in the control group of healthy, fertile, ROS-negative donors, with 51 being over-expressed, whereas 2 were under-expressed (Table S2). Regarding protein abundance in this group, 25 proteins were highly abundant, 110 were of medium abundance, 143 were of low abundance, and 245 were of very low abundance.

### 3.3. Functional Enrichment and Pathway Analysis Highlight Oxidative Stress-Linked Immune and Cellular Dysregulation

Table 2 and Table 3 present the analysis conducted using the DAVID software (https://david.ncifcrf.gov, accessed on 15 June 2024) for both groups (fertile donors with ROS-positive semen and patients diagnosed with secondary infertility). In fertile donors experiencing high oxidative stress, over-expressed proteins were found to be associated with antioxidant activity and immune responses. Conversely, under-expressed proteins were detected to be involved in protein folding, stabilization, binding, and energy metabolism. In patients with secondary infertility, over-expressed proteins were identified to be associated with inflammatory and immune responses, energy metabolism, and cellular stability. Conversely, under-expressed proteins were related to sperm cell motility and capacitation. These unique proteins are involved in protein binding, cell adhesion, and extracellular matrix organization.

Table 4 and Table 5 summarize the result of the IPA analysis. Both comparisons—between control group and ROS-positive donors, as well as control group and secondary infertility patients—showed how oxidative stress influences immune responses and cellular signaling pathways. Important shared pathways, like acute phase response signaling and immune cell trafficking, highlight the role of oxidative stress in activating the immune system and promoting inflammation. Both groups also demonstrated significant involvement in the development of the hematological system, indicating that oxidative stress has a widespread impact on blood and immune cell functions, which is vital for reproductive health. The analyses revealed common disruptions in cellular functions, including cellular movement, cell-to-cell signaling and free radical scavenging, all of which are essential for sperm function and male fertility.

**Figure 3:**
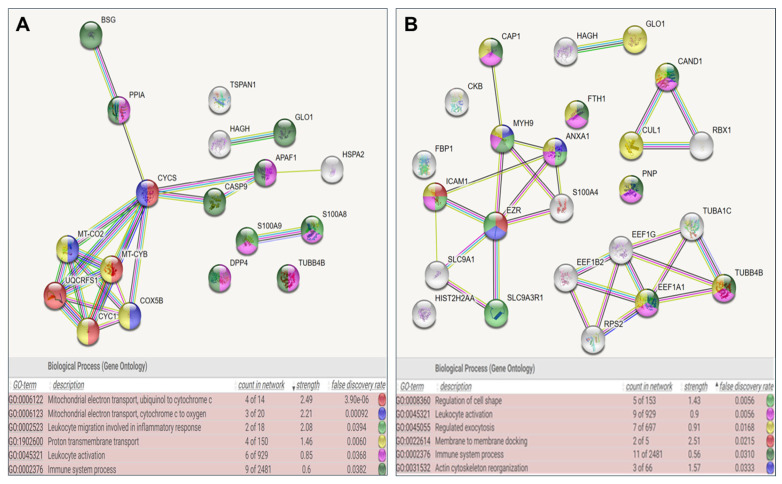
STRING analysis represents the Protein–Protein Interaction network of differentially expressed proteins. (**A**) Fertile donors with ROS-positive versus healthy donors ROS-negative and (**B**) Patients with secondary infertility versus healthy fertile donors ROS-negative. Nodes represent individual proteins, and edges represent interactions between them. Known interactions (from curated databases and experimental evidence) are depicted with solid blue and pink lines, while predicted interactions (gene neighborhood, gene fusions, and gene co-occurrence) are shown with green, red, and blue lines, respectively. Additional associations derived from text mining, co-expression, and protein homology are illustrated using yellow, black, and light purple lines, respectively. The network represents interactions among proteins associated with oxidative stress and infertility, highlighting significant molecular pathways.

Additionally, Table 4 and Table 5 highlight pathways such as mitochondrial electron transport, immune system activation, and oxidative stress-related mechanisms. Key proteins in this group include CYCS, MT-CO2, UQCRFS1, and others implicated in cellular energy metabolism and inflammation. The analysis of patients with secondary infertility reveals distinct and overlapping interactions compared to group 1, focusing on cytoskeletal organization, regulated exocytosis, leukocyte activation, and cell shape regulation. Proteins such as EZR, MYH9, and S100A4 play critical roles in cytoskeletal dynamics and cellular motility, processes associated with sperm function and fertility. Enriched Gene Ontology (GO) biological processes are displayed in Figure 3, ranked by statistical significance (false discovery rate, FDR). Shared pathways include leukocyte activation and immune system processes, suggesting a potential link between oxidative stress, immune dysregulation, and infertility mechanisms. Key proteins such as C3 and SERPINA3 have emerged as potential biomarkers or therapeutic targets in patients with secondary infertility and warrant further investigation (Table 6).

## 4. Discussion

Seminal plasma proteins, crucial for sperm health, motility, fertilization, interaction with the female reproductive system, and genetic material conveyance, also protect against oxidative damage, making it essential to understand the impact of oxidative stress on male fertility. While previous seminal plasma proteomic studies have advanced our understanding of male infertility, they have not sufficiently explored oxidative stress as a mechanistic contributor to secondary infertility. Martins et al. investigated the proteomic profiles of seminal plasma in men with primary and secondary infertility, identifying unique protein biomarkers for each group [10]. While this study established baseline differences in protein expression, it did not consider oxidative stress as a potential driver of these infertility types. Similarly, Wang et al. looked into the roles of seminal extracellular vesicles in sperm function, particularly their effects on motility, capacitation, and the acrosome reaction [22]. Although this research highlighted how seminal extracellular vesicles impact sperm function, it did not examine the influence of oxidative stress on seminal plasma composition or its specific connections to secondary infertility. Dash et al. investigated the effects of COVID-19 recovery on sperm proteomics, revealing how viral exposure can alter sperm parameters and proteomic profiles [23]. In contrast, our study specifically focuses on the proteomic changes in seminal plasma associated with oxidative stress in secondary infertility.

In this study, we investigated whether high oxidative stress is a risk factor for secondary infertility. We hypothesized that untreated oxidative stress could be associated with long-term infertility. This association is important as it enables us to encourage lifestyle changes that reduce oxidative stress and offer antioxidant treatments to avoid the progression to secondary infertility. We utilized our proteomics data on male infertility which compared the proteomic profiles of healthy fertile donors without oxidative stress to those of fertile donors experiencing high oxidative stress in their semen. Additionally, the proteomic profile of patients with secondary infertility was compared to that of healthy fertile donors without oxidative stress. We excluded cases with female factor infertility, chronic diseases and varicocele.

Our findings reveal that DEPs in both fertile donors with high oxidative stress and patients with secondary infertility are involved in overlapping biological pathways and molecular functions. These include acute-phase response signaling, immune cell trafficking, organismal injury and abnormalities, cellular movement, cell-to-cell communication, and hematological system development. The convergence of these pathways suggests that oxidative stress triggers a core set of molecular disturbances that impair immune regulation, cellular integrity, and systemic processes essential for fertility. This overlap highlights oxidative stress as a central contributor to reproductive dysfunction in both groups, disrupting key mechanisms of immune and cellular homeostasis. The molecular function of free radical scavenging was activated in the group of fertile donors with ROS-positive semen and in the group of patients with secondary infertility, suggesting that oxidative stress is a cause of this pathology. Additionally, other shared pathways such as acute-phase response signaling, organismal injury and abnormalities, cellular movement, and immune cell trafficking are also associated with oxidative stress, further emphasizing that oxidative stress is associated with secondary infertility.

Based on our findings, we propose a strong association between oxidative stress and secondary infertility. Studies have demonstrated that oxidative stress is inversely associated with normal sperm parameters, including percentage of normal sperm, concentration, and motility, while being positively associated with higher percentages of abnormal spermatozoa [24,25]. The impact of oxidative stress on sperm motility can be attributed to lipid peroxidation, which compromises the solubility of the plasma membrane. This disruption reduces the phosphorylation of axonemal proteins, ultimately leading to sperm immobilization [26]. Additionally, oxidative stress has also been implicated in SDF, an important marker of sperm nuclear integrity. Elevated SDF levels have been associated with impaired embryo development and lower success rates in assisted reproduction, as indicated by Conflitti et al., who recently demonstrated that high post-selection SDF significantly increases the risk of embryo arrest and non-viability, underscoring the downstream effects of oxidative damage beyond conventional semen parameters [27]. A decreased sperm count may result from prolonged exposure of the seminiferous epithelium to elevated oxidative stress levels caused by immature or abnormal spermatozoa. This oxidative damage can harm the seminiferous tubules, resulting in testicular atrophy and a subsequent decline in gamete production.

Our comprehensive analysis of proteins within each biological pathway and process aimed to identify potential markers of future infertility and associated comorbidities in patients with secondary infertility. Notably, the C3 protein and SERPINA3 were consistently present in all specified biological pathways and molecular functions, particularly the free radical scavenging pathway, showing a positive fold-change in expression, indicating their overexpression. The C3 protein is a glycoprotein and an integral component of the complement immune system, encoded by the *C3* gene on chromosome 19 [28,29]. Elevated C3 levels typically indicate inflammation [30,31]. C3 has been implicated in male infertility as an indicator of underlying inflammation or oxidative stress. C3 is normally present only at very low concentrations in healthy semen; however, it is elevated in certain infertile conditions [32]. For example, men with anti-sperm antibodies show significantly higher seminal C3 levels than fertile controls, reflecting local complement activation [33]. Unchecked complement activity can adversely affect fertility: mice lacking the complement regulator CSMD1 (CUB and Sushi Multiple Domains 1) exhibit excessive C3 deposition in the testes with associated tissue degeneration and subfertility [34]. Conversely, C3 also plays a physiological role in reproduction C3b binding via sperm. CD46 has been shown to facilitate sperm–oocyte interactions in mammals [32], underscoring a nuanced role for C3 in sperm function. SERPINA3, a serine protease inhibitor from the serpin family, modulates inflammatory and oxidative stress pathways [35,36]. Further, SERPINA3 inhibits neutrophil proteases (e.g., cathepsin G) and protects tissues from proteolytic damage during inflammation [37,38]. Previous studies demonstrated that proteins from the serpin family, specifically SERPINA 5, are disrupted in patients with varicocele-associated infertility [39] and spinal cord injury infertility [40]. In line with these human findings, animal models also underscore the relevance of these proteins: mice deficient in surfactant protein D (an immune regulator) display an upregulation of testicular SERPINA3, alongside anti-inflammatory cytokines as a compensatory response to inflammatory challenge [41].

Collectively, our converging evidence from gene and protein expression analyses, large-scale proteomic surveys, and functional studies in both human and animal models provides the first demonstration of an association between C3, SERPINA3, and secondary male infertility. These associations are consistently observed across independent datasets and are biologically plausible given the well-established roles of C3 and SERPIN family in oxidative stress and inflammation mechanisms known to impair male reproductive function. While these findings are biologically plausible and supported by prior literature, we acknowledge that our current study does not include direct functional characterization or protein-level validation (e.g., by immunoblotting or ELISA) of C3 or SERPINA3. Therefore, our suggestion that these proteins may serve as potential biomarkers should be considered preliminary. Future studies are warranted to validate their expression patterns in larger cohorts and to investigate their mechanistic roles in oxidative stress-related infertility. The availability of commercially validated antibodies for both proteins may facilitate such targeted translational studies.

Despite our results, it is crucial to acknowledge certain limitations that may have influenced our findings. The most significant limitation of our study is that it is an association study rather than a causative one. While our data reveals a correlation between oxidative stress and secondary infertility, this design does not establish a direct cause-and-effect relationship. An association study provides valuable insights into patterns and relationships between variables but lacks the ability to confirm whether one variable directly causes changes in another. The lack of temporal data demonstrating whether oxidative stress precedes or results from infertility further limits our ability to draw causal inferences. Additionally, this study does not include interventional data, such as the use of antioxidant treatments to reduce oxidative stress and observe subsequent effects on fertility outcomes. Such interventional studies, along with longitudinal research, would be necessary to confirm whether oxidative stress is a primary factor contributing to secondary infertility or if it is a byproduct of other underlying mechanisms. The relatively small sample size may also limit the generalizability of the findings.

Importantly, our analysis was performed on pooled seminal plasma samples generated under standardized experimental conditions in the original studies, allowing for robust pathway enrichment and biological interpretation. Although individual-level multivariate analyses, such as principal component analysis (PCA), could further strengthen cohort discrimination, the consistent enrichment of oxidative stress-related immune, inflammatory, and cellular pathways across comparisons supports the biological plausibility and relevance of the observed proteomic signatures. The study relies on proteomic analysis; while this is comprehensive, it may not capture all potential biomarkers or mechanisms involved in secondary infertility. Additionally, potential confounding factors such as lifestyle, environmental exposures, and genetic predispositions were not controlled for, which could influence the results. While we minimized confounders like female infertility, smoking, and hormonal imbalances, residual confounders might still exist, preventing definitive conclusions about causation from being made.

Future longitudinal studies that include a broader range of variables and track men with elevated oxidative stress over time could confirm whether oxidative stress directly contributes to infertility. Interventional trials using antioxidant therapies could also provide causal evidence by demonstrating whether reducing oxidative stress improves fertility outcomes. Until such studies are conducted, our results should be interpreted as evidence of an association, providing a foundation for future research into the role of oxidative stress in secondary infertility.

## 5. Conclusions

Our proteomic findings strongly suggest that oxidative stress plays a significant role in secondary infertility. The overexpression of key proteins such as C3 and SERPINA3 reinforces the connection between oxidative stress and inflammatory responses in male fertility and highlights them as potential biomarkers for future diagnosis and treatment. While our study highlights oxidative stress as a critical factor, further research is needed to establish causality and explore the efficacy of antioxidant-based interventions. These findings also underscore the broader implications of oxidative stress on male health, emphasizing the importance of early detection, lifestyle modifications, and preventive measures to address its far-reaching consequences.

## Figures and Tables

**Table 1:** Results from semen analysis, sperm DNA fragmentation (SDF), and intracellular reactive oxygen species (ROS) testing in both groups.

Semen Parameters	Fertile Donors ROS Negative (*n* = 10)	Fertile Donors ROS Positive (*n* = 10)	Patients with Secondary Infertility (*n* = 10)
Sperm concentration (10^6^/mL)	52.0 (66.8–38.2)	25.6 (57.9–12.6)	24.5 (66.9–11.9)
Sperm motility (%)	47.00 (50.5–40)	39 (56.5–20)	30 (42– 8)
Normal sperm morphology (%)	6.00 (8.5–5)	4 (6.5–2)	4 (5–1.5)
ROS levels (RLU/s/10^6^ sperm)	60.60 (71.9–28.25)	1237.7 (3820.75–139.75)	350.7 (879.75–100.2)
SDF (%)	9.3 (9.5–8.9)	19 (20.9–7.8)	16.5 (18.45–6.75)

Results are presented as the median with the 25th and 75th percentile. RLU: relative light units.

**Table 2:** Results of DAVID software analysis: Differentially expressed proteins and their biological processes and molecular functions in fertile donors with ROS-positive semen.

**Biological Processes**	
**Over-expressed Proteins**	Negative regulation of endopeptidase activity (SERPINA1, SERPINA3, SERPINB6, and SERPING1); **Acute-phase response** (A2M, APCS, HP, SERPINA1, and SERPINA3); **Cellular oxidant detoxification** (S100A9, ALB, HP, and PRDX4); **Innate immunity response** (S100A9, C3, LCN2, and SERPING1)
**Under-expressed Proteins**	**Protein folding** (APCS, CCT4, HSP90AA1, HSP90AB1, HSP90B1, and PPIA); **Positive regulation of telomerase activity** (CCT4, HSP90AA1, and HSP90AB1); **Response to cold** (HSP90AA1, HSPA2, and LPL); **Response to unfolded protein** (HSP90AA1, HSP90AB1, and HSPA2); **Protein stabilization** (CCT4, HSP90AA1, HSP90AB1, and TSPAN1); **Response to xenobiotic stimulus** (HSP90AA1, HSP90AB1, and LPL)
**Molecular Functions**	
**Over-expressed Proteins**	**Serine protease inhibitor** (APP, SPINT1, SERPINA3, and SERPINA4); **Antioxidant activity** (S100A9, ALB, and HP); **Protease binding** (CTSD, DPP4, KLK3, and PGC); **Antioxidant** (S100A9, S100A9, ALB, and HP); **Zinc ion binding** (S100A9, ALB, QPCT, and GLO1)
**Under-expressed Proteins**	**Unfolded protein binding** (APCS, CCT4, HSP90AA1, HSP90AB1, HSP90B1, HSPA2, PPIA, and TUBB4B); **Protein binding involved in protein folding, Chaperone** (CCT4, HSP90AA1, HSP90AB1, HSP90B1, and HSPA2); **ATPase activity** (CCT4, HSP90AA1, HSP90AB1, HSP90B1, and HSPA2); **Disordered domain specific binding** (HSP90AA1, HSP90AB1, and HSPA2); **Tau protein binding** (HSP90AA1, HSP90AB1, and HSPA2); **RNA binding** (CCT4, HSP90AA1, HSP90AB1, HSP90B1, PPIA, and YWHAZ); **Ion channel binding** (HSP90AA1, HSP90AB1, and YWHAZ); **ATP binding** (CCT4, HSP90AA1, HSP90AB1, HSP90B1, and HSPA2); **GTP binding** (HSP90AA1, HSP90AB1, and TUBB4B); **Identical protein binding (3), Protein homodimerization activity** (DPP4, HSP90AA1, HSP90AB1, and LPL)

**Table 3:** Results of DAVID software analysis: Differentially expressed proteins and their biological processes and molecular functions in patients with secondary infertility.

**Biological Processes**	
**Over-expressed Proteins**	**Serine-type endopeptidase inhibitor activity** (APP, ANXA2, SPINT1, SERPINA3, and ERPINA4); **Cell adhesion** (APP, LAMA5, MYH9, NPNT, OLFM4, and PTPRS); **Actin cytoskeleton reorganization** (ANXA1, EZR, MYH9, and RHOA); **Regulation of cell shape** (ANXA1, EZR, MYH9, and RHOA); **Proteolysis** (C3, LGMN, PRCP, and TMPRSS2); **Inflammatory response** (ANXA1, C3, C4A, and SERPINA3); **Innate immunity response** (ANXA1, C3, and C4A)
**Under-expressed Proteins**	**Coagulation** (SEMG1 and SEMG2); **Positive regulation of serine-type endopeptidase activity** (SEMG1 and SEMG2); **Negative regulation of flagellated sperm motility** (SEMG1 and SEMG2); **Sperm capacitation** (SEMG1 and SEMG2); **Antibacterial humoral response** (SEMG1 and SEMG2)
**Unique Proteins**	**Cell adhesion** (APP, LAMA5, MYH9, NPNT, OLFM4, and PTPRS); **Extracellular matrix organization** (APP, NPNT, and SPINT1)
**Molecular Functions**	
**Over-expressed Proteins**	**Serine-type endopeptidase inhibitor activity** (APP, SPINT1, SERPINA3, and SERPINA4); **Protease** (APP, LGMN, PRCP, and TMPRSS2); **Integrin binding** (APP, LAMA5, LCP1, MYH9, and NPNT); **Cadherin binding** (EZR, HSPA5, LDHA, MYH9, and OLFM4); **Actin filament binding** (EZR, LCP1, and MYH9); **GTP binding** (RAB27A, EEF1A1, RHOA, TUBA1C, and TUBB4B); **GTPase activity** (RAB27A, EEF1A1, RHOA, TUBA1C, and TUBB4B); **Calcium ion binding** (ANXA1, ANXA2, HSPA5, LCP1, and NPNT)
**Under-expressed Proteins**	**Zinc ion binding** (SEMG1 and SEMG2)
**Unique Proteins**	**Integrin binding** (APP, MYH9, and NPNT); **Protein binding** (RAB27A, EZR, HSPA5, MYH9, and RHOA); **Identical protein binding** (APP, FTH1, FBP1, and MYH9)

**Table 4:** Results of IPA software analysis: Fertile healthy donors with ROS negative vs. fertile donors with ROS positive.

Top Canonical Pathways	*p*-Value	Overlap
Acute Phase Response Signaling	3.82 × 10^−7^	32.8% (6/185)
Role of PKR in Interferon Induction and Antiviral Response	2.10 × 10^−6^	35.3% (5/136)
Prostate Cancer Signaling	2.98 × 10^−5^	3.5% (4/114)
Neuroprotective Role of THOP1 in Alzheimer’s Disease	3.54 × 10^−5^	33.3% (4/120)
eNOS Signaling	1.06 × 10^−4^	2.5% (4/159)
Top Diseases and Bio Functions	*p*-value range	Proteins
Cancer	8.37 × 10^−8^–8.27 × 10^−11^	18
Endocrine System Disorders	7.14 × 10^−8^–8.27 × 10^−11^	13
Organismal Injury and Abnormalities	8.37 × 10^−8^–8.27 × 10^−11^	28
Reproductive System Disease	8.37 × 10^−8^–8.27 × 10^−11^	13
Respiratory Disease	7.14 × 10^−8^–3.38 × 10^−9^	6
Molecular and Cellular Functions		
Cellular Movement	7.91 × 10^−9^–1.31 × 10^−2^	21
Cell-To-Cell Signaling and Interaction	7.99 × 10^−8^–9.72 × 10^−9^	5
Post-Translational Modification	1.17 × 10^−8^–1.17 × 10^−8^	6
Protein Folding	1.17 × 10^−8^–1.17 × 10^−8^	6
Free Radical Scavenging	4.99 × 10^−8^–4.99 × 10^−8^	11
Physiological System Development and Function		
Immune Cell Trafficking	9.72 × 10^−9^–1.31 × 10^−12^	19
Hematological System Development and Function	7.99 × 10^−8^–4.70 × 10^−11^	20

**Table 5:** Results of IPA software analysis: Fertile healthy donors with ROS negative vs. patients with secondary infertility.

Top Canonical Pathways	*p*-value	Overlap
LXR/RXR Activation	8.29 × 10^−7^	4.1% (5/123)
FXR/RXR Activation	9.34 × 10^−7^	4.0% (5/126)
Acute Phase Response Signaling	6.16 × 10^−6^	2.7% (5/185)
BAG2 Signaling Pathway	2.33 × 10^−4^	3.6% (3/84)
Complement System	1.27 × 10^−3^	5.4% (2/37)
Top Diseases and Bio Functions	*p*-value range	Proteins
Infectious Diseases	1.49 × 10^−3^–6.60 × 10^−9^	17
Organismal Injury and Abnormalities	2.85 × 10^−3^–6.60 × 10^−9^	34
Dermatological Diseases and Conditions	2.85 × 10^−3^–1.19 × 10^−7^	29
Inflammatory Response	2.85 × 10^−3^–1.34 × 10^−7^	22
Neurological Disease	2.85 × 10^−3^–1.93 × 10^−7^	31
Molecular and Cellular Functions		
Cellular Movement	2.85 × 10^−3^–3.58 × 10^−9^	21
Cell-To-Cell Signaling and Interaction	2.85 × 10^−3^–1.74 × 10^−6^	18
Cell Death and Survival	2.85 × 10^−3^–7.16 × 10^−6^	20
Cellular Function and Maintenance	2.83 × 10^−3^–1.15 × 10^−5^	21
Free Radical Scavenging	1.48 × 10^−3^–1.54 × 10^−5^	8
Physiological System Development and Function		
Immune Cell Trafficking	2.85 × 10^−3^–6.42 × 10^−9^	15
Hematological System Development and Function	2.85 × 10^−3^–1.91 × 10^−8^	18
Behavior	5.83 × 10^−6^–5.83 × 10^−6^	4
Renal and Urological System Development and Function	2.47 × 10^−3^–8.79 × 10^−6^	7
Organismal Functions	2.35 × 10^−5^–2.35 × 10^−5^	5

**Table 6:** Bioinformatics findings indicate altered expression of C3 and SERPINA3 proteins in the seminal plasma of patients with secondary infertility.

Proteins	Biological Processes	Molecular Functions	Expression Fold Change
C3	Proteolysis, Inflammatory response, Innate immunity response	Protease activity	9.867
SERPINA3	Serine-type endopeptidase inhibitor activity, Inflammatory response, Innate immunity response	Serine-type endopeptidase inhibitor activity, Antioxidant activity	8.025

## Data Availability

Data from our study are available from the corresponding author upon reasonable request.

## Supplementary Materials

The following supporting information can be downloaded at: https://www.mdpi.com/article/10.3390/jcm15031173/s1, Table S1: Differentially expressed proteins with normalized spectral abundance factor ratios in healthy fertile donors without oxidative stress vs. fertile donors with high oxidative stress. Table S2: Differentially expressed proteins with normalized spectral abundance factor ratios in healthy fertile donors without oxidative stress vs. patients with secondary infertility.
