# Supplementary material for: Proteomic Profiles of Seminal Plasma in Individuals with Secondary Infertility: Insights into the Involvement of Oxidative Stress
Source: J Clin Med. 2026 Feb 2;15(3):1173. doi: 10.3390/jcm15031173 (PMC12898425; doi:10.3390/jcm15031173)
Supplement: Supplementary file 1 [file jcm-15-01173-s001.zip › jcm-4068302-supplementary.pdf]

Supplementary Table S1. Differentially expressed proteins (DEPs) with normalized spectral abundance factor (NSAF) ratios.

Group 1: Healthy fertile donors without oxidative stress.

Group 2: Fertile donors with high oxidative stress.

Protein	accession	Average SC		Abundance		NSAF ratio	T-Test	Expression
		Group 1	Group 2	Group 1	Group 2	Group 2/Group 1	P-value	
**T-complex protein 1 subunit delta isoform a**	**38455427**	2.7	0	VL	ni	0.00	0.00011	Unique to Lane 1
**serine protease inhibitor Kazal-type 2 isoform 1 precursor**	**413081531**	9.7	1.3	L	VL	0.12	0.00297	Underexpressed
**tubulin beta-4B chain**	**5174735**	13.7	1.7	L	VL	0.12	0.00703	Underexpressed
**nucleobindin-2 isoform X1**	**578820554**	22.7	3.0	M	VL	0.13	0.00677	Underexpressed
**peptidyl-prolyl cis-trans isomerase A**	**10863927**	10.3	3.0	L	VL	0.21	0.00463	Underexpressed
**lipoprotein lipase precursor**	**4557727**	35.7	7.0	M	VL	0.23	0.01928	Underexpressed
**semenogelin-2 precursor**	**4506885**	1321.0	409.0	H	H	0.26	0.00023	Underexpressed
**semenogelin-1 preproprotein**	**4506883**	698.7	236.3	H	H	0.26	0.00074	Underexpressed
**heat shock-related 70 protein 2**	**13676857**	46.3	25.3	M	M	0.30	0.00554	Underexpressed
**14-3-3 protein zeta/delta isoform X2**	**530389317**	20.3	6.3	M	VL	0.32	0.02786	Underexpressed
**heat shock protein HSP 90-beta isoform c**	**431822408**	17.7	10.7	L	L	0.35	0.00026	Underexpressed
**heat shock protein HSP 90-alpha isoform 1**	**153792590**	70.0	43.0	M	M	0.36	0.01023	Underexpressed
**endoplasmin precursor**	**4507677**	16.7	9.0	L	L	0.37	0.00602	Underexpressed
**gastricsin isoform 1 preproprotein**	**4505757**	49.3	24.7	M	M	0.50	0.00149	Underexpressed
**prostate-specific antigen isoform 1 preproprotein**	**4502173**	321.0	225.0	H	H	0.66	0.00839	Underexpressed
**serum albumin preproprotein**	**4502027**	741.0	1225.3	H	H	1.51	0.00583	Overexpressed
**zinc-alpha-2-glycoprotein precursor**	**4502337**	206.3	272.0	H	H	1.60	0.03459	Overexpressed
**epididymal secretory protein E1 precursor**	**5453678**	85.7	146.7	H	H	1.62	0.03894	Overexpressed
**alpha-2-macroglobulin isoform X1**	**578822814**	17.0	40.0	L	M	2.01	0.01423	Overexpressed
**cysteine-rich secretory protein 1 isoform 1 precursor**	**25121982**	27.3	51.0	M	M	2.05	0.00951	Overexpressed
**glycodelin precursor**	**65507501**	25.0	54.7	M	M	2.19	0.00242	Overexpressed
**neprilysin isoform X1**	**578807443**	15.0	31.0	L	M	2.22	0.03895	Overexpressed

**alpha-1-antitrypsin precursor**	**189163532**	129.3	196.7	H	H	2.23	0.02790	Overexpressed
**cathepsin D preproprotein**	**4503143**	39.7	65.3	M	M	2.25	0.00977	Overexpressed
**cartilage acidic protein 1 isoform B precursor**	**330688397**	23.0	44.0	M	M	2.47	0.00449	Overexpressed
**serpin B6 isoform a**	**41152086**	8.0	15.3	L	L	2.68	0.00424	Overexpressed
**serum amyloid P-component precursor**	**4502133**	6.0	15.3	VL	L	2.69	0.00538	Overexpressed
**cytochrome c**	**11128019**	2.7	12.0	VL	L	2.84	0.00550	Overexpressed
**dipeptidyl peptidase 4**	**18765694**	18.3	47.0	L	M	3.10	0.02836	Overexpressed
**alpha-1-antichymotrypsin precursor**	**50659080**	40.7	106.0	M	H	3.23	0.00023	Overexpressed
**plasma protease C1 inhibitor precursor**	**73858570**	17.7	60.7	L	M	3.30	0.00049	Overexpressed
**peroxiredoxin-4 precursor**	**5453549**	3.0	10.0	VL	L	3.39	0.00099	Overexpressed
**protein S100-A9**	**4506773**	4.0	22.7	VL	M	3.77	0.01707	Overexpressed
**sialate O-acetylesterase isoform 1 precursor**	**24850115**	6.7	24.3	VL	M	3.80	0.00592	Overexpressed
**IgGFc-binding protein precursor**	**154146262**	16.3	77.7	L	M	5.98	0.00010	Overexpressed
**haptoglobin isoform 2 preproprotein**	**186910296**	1.7	10.3	VL	L	9.03	0.00349	Overexpressed
**olfactomedin-4 precursor**	**32313593**	3.0	34.3	VL	M	9.37	0.00015	Overexpressed
**maltase-glucoamylase, intestinal isoform X1**	**578814724**	3.3	29.3	VL	M	9.55	0.00024	Overexpressed
**tetraspanin-1**	**21264578**	0.3	3.0	VL	VL	11.00	0.00074	Overexpressed
**glutaminyl-peptide cyclotransferase precursor**	**6912618**	0.3	3.0	VL	VL	12.77	0.00074	Overexpressed
**complement C3 precursor**	**115298678**	2.7	62.3	VL	M	17.22	0.00210	Overexpressed
**lactoylglutathione lyase**	**118402586**	1.3	17.0	VL	L	17.92	0.00737	Overexpressed
**neutrophil gelatinase-associated lipocalin precursor**	**38455402**	0.7	19.3	VL	L	37.47	0.00445	Overexpressed
**polymeric immunoglobulin receptor isoform X1**	**530366266**	0.3	28.0	VL	M	73.92	0.00350	Overexpressed

Supplementary Table S2. Differentially expressed proteins (DEPs) with normalized spectral abundance factor (NSAF) ratios.

Group 1: Healthy fertile donors without oxidative stress.

Group 2: Patients with secondary infertility.

Protein	accession	Average SC		Abundance		NSAF ratio	T-Test
		Group 1	Group 2	Group 1	Group 2	Group 2/Group 1	P-value
**semenogelin-2 precursor**	**4506885**	1321.3	1310.0	H	H	0.54	0.001388
**semenogelin-1 preproprotein**	**4506883**	698.7	742.0	H	H	0.59	0.009700
**serotransferrin precursor**	**4557871**	63.3	214.3	M	H	1.52	0.009638
**alpha-1-antichymotrypsin precursor**	**50659080**	40.7	144.0	M	H	1.90	0.000762
**elongation factor 1-alpha 1**	**4503471**	20.3	56.7	M	M	2.02	0.000280
**78 glucose-regulated protein precursor**	**16507237**	8.7	44.3	L	M	2.24	0.048217
**tubulin beta-4B chain**	**5174735**	13.7	44.0	L	M	2.27	0.011258
**creatine kinase B-type**	**21536286**	15.7	53.0	L	M	2.29	0.001589
**cytosolic non-specific dipeptidase isoform X2**	**530414265**	8.7	32.0	L	M	2.37	0.040321
**transmembrane protease serine 2 isoform 2**	**205360943**	8.0	34.0	L	M	2.46	0.004135
**annexin A1**	**4502101**	7.7	33.0	VL	M	2.75	0.012865
**kallistatin isoform 2 precursor**	**21361302**	9.0	41.0	L	M	2.75	0.025892
**purine nucleoside phosphorylase**	**157168362**	4.7	24.0	VL	M	2.79	0.010691
**alpha-2-antiplasmin isoform X1**	**578840157**	11.0	46.0	L	M	2.90	0.016517
**receptor-type tyrosine-protein phosphatase S isoform X1**	**530425335**	5.0	25.3	VL	M	2.92	0.019307
**annexin A2 isoform 1**	**50845388**	4.7	20.0	VL	M	3.06	0.017794
**cullin-associated NEDD8-dissociated protein 1**	**21361794**	4.3	26.3	VL	M	3.10	0.021171
**complement C4-A isoform 1 preproprotein**	**67190748**	7.3	49.7	VL	M	3.19	0.031549
**L-lactate dehydrogenase A chain isoform 1**	**5031857**	5.0	25.3	VL	M	3.25	0.000981
**ras-related protein Rab-27A isoform X2**	**530406261**	3.0	18.0	VL	L	3.28	0.005380
**beta-hexosaminidase subunit alpha preproprotein**	**189181666**	6.3	34.0	VL	M	3.50	0.001906

**ezrin**	**21614499**	2.0	9.7	VL	L	3.50	0.004375
**olfactomedin-4 precursor**	**32313593**	3.0	25.0	VL	M	3.53	0.000465
**beta-galactosidase isoform b**	**119372312**	3.0	22.0	VL	M	3.57	0.002897
**tubulin alpha-1C chain**	**14389309**	9.0	40.3	L	M	3.60	0.001176
**maltase-glucoamylase, intestinal isoform X1**	**578814724**	3.3	29.0	VL	M	3.69	0.010355
**heat shock cognate 71 protein isoform X1**	**578822169**	3.0	27.7	VL	M	4.02	0.002540
**plastin-2 isoform X2**	**530402335**	10.0	65.7	L	M	4.11	0.005525
**legumain preproprotein**	**56682962**	3.0	21.0	VL	M	4.80	0.004225
**alpha-1-acid glycoprotein 2 precursor**	**4505529**	1.7	13.0	VL	L	4.87	0.005891
**lactoylglutathione lyase**	**118402586**	1.3	11.0	VL	L	5.48	0.002352
**alpha-1B-glycoprotein precursor**	**21071030**	1.3	17.0	VL	L	6.11	0.002509
**laminin subunit alpha-5 isoform X1**	**578835999**	1.7	27.0	VL	M	7.06	0.021524
**lipocalin-15 precursor**	**42714611**	0.7	10.0	VL	L	8.39	0.001023
**dipeptidase 3 isoform a precursor**	**193211608**	0.7	13.3	VL	L	10.10	0.002087
**complement C3 precursor**	**115298678**	2.7	92.0	VL	H	15.37	0.004311
**programmed cell death 6-interacting protein isoform 1**	**22027538**	0.7	21.0	VL	M	15.60	0.025954
**adenylyl cyclase-associated protein 1**	**5453595**	0.3	11.3	VL	L	22.01	0.001582
**polymeric immunoglobulin receptor isoform X1**	**530366266**	0.3	29.0	VL	M	45.00	0.003562
**histone H2A type 2-A**	**4504251**	0.3	18.0	VL	L	49.22	0.005117
**ferritin heavy chain**	**56682959**	0.0	2.0	ni	VL	Group 8 only	0.000023
**fructose-1,6-bisphosphatase 1**	**16579888**	0.0	6.7	ni	VL	Group 8 only	0.000851
**nephronectin isoform A precursor**	**296011067**	0.0	6.0	ni	VL	Group 8 only	0.000262
**transforming protein RhoA precursor**	**10835049**	0.0	2.0	ni	VL	Group 8 only	0.000024
**ceruloplasmin precursor**	**4557485**	0.0	9.3	ni	L	Group 8 only	0.000125
**importin-5 isoform X2**	**530423350**	0.0	12.0	ni	L	Group 8 only	0.000152
**lysosomal Pro-X carboxypeptidase isoform 1 preproprotein**	**4826940**	0.0	10.3	ni	L	Group 8 only	0.000232
**heat shock 70 protein 1-like isoform X1**	**530381921**	0.0	8.7	ni	L	Group 8 only	0.000495
**carboxylesterase 5A isoform 1 precursor**	**219521907**	0.0	14.3	ni	L	Group 8 only	0.000841
**kunitz-type protease inhibitor 1 isoform 1 precursor**	**32313599**	0.0	8.0	ni	L	Group 8 only	0.003919
**myosin-9**	**12667788**	0.0	17.0	ni	L	Group 8 only	0.004667

**amyloid beta A4 protein isoform f precursor**	**209915573**	0.0	8.3	ni	L	Group 8 only	0.006708
**alpha-crystallin A chain-like isoform X1**	**578836360**	0.0	51.3	ni	M	Group 8 only	0.045285
**T-complex protein 1 subunit delta isoform a**	**38455427**	0.0	0	0	ni	0.00	0.00000
**serine protease inhibitor Kazal-type 2 isoform 1 precursor**	**413081531**	0.0	0.0	0	VL	0.00	0.00000
**tubulin beta-4B chain**	**5174735**	0.0	0.0	0	VL	0.00	0.00000
**nucleobindin-2 isoform X1**	**578820554**	0.0	0.0	0	VL	0.00	0.00000
**peptidyl-prolyl cis-trans isomerase A**	**10863927**	0.0	0.0	0	VL	0.00	0.00000
**lipoprotein lipase precursor**	**4557727**	0.0	0.0	0	VL	0.00	0.00000
**semenogelin-2 precursor**	**4506885**	0.0	0.0	0	H	0.00	0.00000
**semenogelin-1 preproprotein**	**4506883**	0.0	0.0	0	H	0.00	0.00000
**heat shock-related 70 protein 2**	**13676857**	0.0	0.0	0	M	0.00	0.00000
**14-3-3 protein zeta/delta isoform X2**	**530389317**	0.0	0.0	0	VL	0.00	0.00000
**heat shock protein HSP 90-beta isoform c**	**431822408**	0.0	0.0	0	L	0.00	0.00000
**heat shock protein HSP 90-alpha isoform 1**	**153792590**	0.0	0.0	0	M	0.00	0.00000
**endoplasmin precursor**	**4507677**	0.0	0.0	0	L	0.00	0.00000
**gastricsin isoform 1 preproprotein**	**4505757**	0.0	0.0	0	M	0.00	0.00000
**prostate-specific antigen isoform 1 preproprotein**	**4502173**	0.0	0.0	0	H	0.00	0.00000
**serum albumin preproprotein**	**4502027**	0.0	0.0	H	0.0	0.00	0.00000
**zinc-alpha-2-glycoprotein precursor**	**4502337**	0.0	0.0	H	0.0	0.00	0.00000
**epididymal secretory protein E1 precursor**	**5453678**	0.0	0.0	H	0.0	0.00	0.00000
**alpha-2-macroglobulin isoform X1**	**578822814**	0.0	0.0	L	0.0	0.00	0.00000
**cysteine-rich secretory protein 1 isoform 1 precursor**	**25121982**	0.0	0.0	M	0.0	0.00	0.00000
**glycodelin precursor**	**65507501**	0.0	0.0	M	0.0	0.00	0.00000
**neprilysin isoform X1**	**578807443**	0.0	0.0	L	0.0	0.00	0.00000
**alpha-1-antitrypsin precursor**	**189163532**	0.0	0.0	H	0.0	0.00	0.00000
**cathepsin D preproprotein**	**4503143**	0.0	0.0	M	0.0	0.00	0.00000
**cartilage acidic protein 1 isoform B precursor**	**330688397**	0.0	0.0	M	0.0	0.00	0.00000
**serpin B6 isoform a**	**41152086**	0.0	0.0	L	0.0	0.00	0.00000
**serum amyloid P-component precursor**	**4502133**	0.0	0.0	VL	0.0	0.00	0.00000

**cytochrome c**	**11128019**	0.0	0.0	VL	0.0	0.00	0.00000
**dipeptidyl peptidase 4**	**18765694**	0.0	0.0	L	0.0	0.00	0.00000
**alpha-1-antichymotrypsin precursor**	**50659080**	0.0	0.0	M	0.0	0.00	0.00000
**plasma protease C1 inhibitor precursor**	**73858570**	0.0	0.0	L	0.0	0.00	0.00000
**peroxiredoxin-4 precursor**	**5453549**	0.0	0.0	VL	0.0	0.00	0.00000
**protein S100-A9**	**4506773**	0.0	0.0	VL	0.0	0.00	0.00000
**sialate O-acetylesterase isoform 1 precursor**	**24850115**	0.0	0.0	VL	0.0	0.00	0.00000
**IgGFc-binding protein precursor**	**154146262**	0.0	0.0	L	0.0	0.00	0.00000
**haptoglobin isoform 2 preproprotein**	**186910296**	0.0	0.0	VL	0.0	0.00	0.00000
**olfactomedin-4 precursor**	**32313593**	0.0	0.0	VL	0.0	0.00	0.00000
**maltase-glucoamylase, intestinal isoform X1**	**578814724**	0.0	0.0	VL	0.0	0.00	0.00000
**tetraspanin-1**	**21264578**	0.0	0.0	VL	0.0	0.00	0.00000
**glutaminyl-peptide cyclotransferase precursor**	**6912618**	0.0	0.0	VL	0.0	0.00	0.00000
**complement C3 precursor**	**115298678**	0.0	0.0	VL	0.0	0.00	0.00000
**lactoylglutathione lyase**	**118402586**	0.0	0.0	VL	0.0	0.00	0.00000
**neutrophil gelatinase-associated lipocalin precursor**	**38455402**	0.0	0.0	VL	0.0	0.00	0.00000
**polymeric immunoglobulin receptor isoform X1**	**530366266**	0.0	0.0	VL	0.0	0.00	0.00000
